# Similarities Between Pediatric and General Hospitals Based on Fundamental Attributes of Surgery Including Cases Per Surgeon Per Workday

**DOI:** 10.7759/cureus.21736

**Published:** 2022-01-30

**Authors:** Richard H Epstein, Franklin Dexter, Christian Diez, Brenda G Fahy

**Affiliations:** 1 Anesthesiology, University of Miami Miller School of Medicine, Miami, USA; 2 Anesthesia, University of Iowa, Iowa City, USA; 3 Anesthesiology, University of Florida, Gainesville, USA

**Keywords:** utilization, block time, case scheduling, operating room management, pediatric surgery

## Abstract

Introduction

Operating room (OR) management decision-making at both pediatric and adult hospitals is determined, in large part, by the same fundamental attributes of surgery and other considerations related to case duration prediction. These include the non-preemptive nature of surgeries, wide prediction limits for case duration, and constraints to moving or resequencing cases on the day of surgery. Another attribute fundamentally affecting OR management is the median number of cases a surgeon performs on their OR days. Most adult surgeons have short lists of cases (i.e., one or two cases per day). Similarly, at adult hospitals, growth in caseloads is mostly due to the subset of those surgeons who also operate just once or twice per week. It is unknown if these characteristics of surgery apply to pediatric surgeons and pediatric hospitals as well.

Methods

Our retrospective cohort study included all elective surgical cases performed at the six pediatric hospitals in Florida during 2018 and 2019 (n = 71,340 cases). We calculated the percentages of combinations of surgeon, date, and hospital (lists) comprising one or two cases, or just one case, and determined if the values were statistically >50% (i.e., indicative of “most”). We determined if most of the growth in caseload and intraoperative work relative value units (wRVUs) at the pediatric hospitals between 2018 and 2019 accrued from low-caseload surgeons. Results are reported as mean ± standard error of the mean.

Results

Averaging among the six pediatric hospitals, the non-holiday weekday lists of most surgeons at each facility had just one or two elective cases, inpatient and/or ambulatory (68.1%; p = 0.016 vs. 50%, n = 27,557 lists). Growth in surgical caseloads from 2018 to 2019 was mostly attributable to surgeons who in 2018 averaged ≤2.0 cases per week (76.3% ± 5.4%, p = 0.0085 vs. 50%). Similarly, growth in wRVUs was mostly attributable to these low-caseload surgeons (73.8% ± 5.4%, p = 0.017 vs. 50%).

Conclusions

Like adult hospitals, most pediatric surgeons’ lists of cases consist of only one or two cases per day, with many lists containing a single case. Similarly, growth at pediatric hospitals accrued from low-caseload surgeons who performed one or two cases per week in the preceding year. These findings indicate that hospitals desiring to increase their surgical caseload should ensure that low-caseload surgeons are provided access to the OR schedule. Additionally, since percent-adjusted utilization and raw utilization cannot be accurately measured for low-caseload surgeons, neither metric should be used to allocate OR time to individual surgeons. Since most adult and pediatric surgeons have low caseloads, this is a fundamental attribute of surgery.

## Introduction

Operating room (OR) management decision-making is determined, in large part, by fundamental attributes of surgery and other considerations related to case duration prediction. One of the most important attributes of surgery is that it is non-preemptive: once started, it is rare that a case is stopped and continued on a different day, even if it will take much longer than scheduled. Wide prediction limits for estimated case duration exist due to process variation [1] and parameter uncertainty [2], the latter resulting from small sample sizes due to the many different Current Procedural Terminology (CPT®) or International Classification of Diseases (ICD) Procedure Codes [3-5]. Pooling multiple procedures of different mean durations increases sample size, but at the expense of greater process variation, resulting in comparable prediction interval widths [1,6]. Also relevant are factors that create constraints to moving or resequencing cases on the day of surgery, such as the need for specialized nursing and anesthesia teams (e.g., for thoracic anesthesia) or equipment (e.g., an operating microscope) [7], and the lack of interchangeability of rooms [8].

Surgery's fundamental attributes and implications are the same for pediatric and adult facilities. Non-preemptive considerations are identical, regardless of the patient’s age. Also, there is no reason to think that process variation would be less impactful in children, and parameter uncertainty has been shown to be a larger problem at pediatric hospitals than at adult hospitals [9,10]. Thus, the various Bayesian and other mathematical methods that have been developed for adult patients to manage rare combinations of procedures apply equally to children [11,12].

Another potential source of variability affecting OR management decision-making is the median number of cases that a surgeon performs on the days they operate [13,14]. Recent studies in both Iowa and Florida have demonstrated that most surgeons' daily elective lists of cases consist of only one or two cases [15,16] and that most surgical growth occurs from surgeons who average only one or two cases per week [17,18]. By "most," we mean that the 99% lower confidence interval (CI) of the mean percentage of lists comprising one or two cases was >50%. One important implication of these findings for hospitals is that percent utilization, either adjusted (including turnover times) or raw (without turnover times) [19], is an unreliable metric for planning individual surgeons' block time [14,15]. Rather, OR time for their cases should be allocated based on maximizing the efficiency of use of OR time of their service [19-21]. A second important implication is that surgical governance committees at hospitals interested in surgical growth need to ensure that low-caseload surgeons have access to the OR schedule and should not focus their efforts on meeting the demands of the high-caseload surgeons [17,22].

However, the facilities we studied previously [15-18] were mostly all adult hospitals or surgery centers. For example, in the 2018 and 2019 datasets from Florida, 94.1% of the cases involved adult patients (i.e., ≥18 years old). In the current study, we reanalyzed our Florida dataset to answer two important questions related to the generalizability of the findings to pediatric hospitals: a) do most pediatric surgeons have lists of one or two cases on the days they operate? and b) does the strategic objective relevant to surgical growth also apply to pediatric hospitals? Whether the same conclusions would be reached is unknown because much of pediatric surgery involves simple, relatively brief procedures in otherwise healthy patients (e.g., myringotomy tubes, tonsillectomies and adenoidectomies, hernia and hydrocele repairs, or minor urological procedures). If our previous conclusions were confirmed for pediatric hospitals and pediatric surgeons as well, it would extend the list of fundamental attributes of surgery to include the fact that most surgeons have short lists of cases (i.e., one or two cases per day) and that most surgical growth accrues from the combined contribution of low-caseload surgeons.

## Materials and methods

We retrospectively analyzed publicly available data from the Agency for Health Care Administration (AHCA) for all patients undergoing ambulatory or inpatient surgery on regular workdays between January 1, 2018, and December 31, 2019, at all ambulatory surgical facilities and non-federal hospitals in Florida. Because the public data only include the year and quarter of the encounter (for outpatients) or admission (for inpatients), we entered into a data use agreement with AHCA to obtain the actual dates, necessary to perform the current study. The Institutional Review Boards of the University of Miami (determination letter September 8, 2020) and the University of Florida (IRB202002442) approved this project as non-human subjects research and exempt, respectively. AHCA disclaims any responsibility for the results and conclusions of the study.

We followed the guidelines from the Equator Network guideline: "The Strengthening the Reporting of Observational Studies in Epidemiology (STROBE) Statement: guidelines for reporting observational studies." We applied the same methods of analysis that are described in detail in our two previous publications related to surgeon lists and surgical growth in Florida [16,18], as summarized in the sections below.

Fundamental attributes of surgery

Some fundamental attributes of surgery and other considerations related to case duration prediction are summarized in Table 1.

**Table 1:** Similarities between pediatric and adult hospitals in surgery and operating room management decision-making ^a^Surgery contrasts with clinic appointments. If an appointment cannot be completed in the scheduled time, the visit can be terminated and a follow-up appointment made within a few days. In contrast, if a thoracic surgeon is doing a lung resection and the dissection is unexpectedly difficult, there is no alternative but to complete the case, regardless of how long it takes ^b^There are thousands of procedures in the Current Procedural Terminology or International Classification of Diseases Procedure Codes, and cases often consist of more than one procedure [1-5,11,12] ^c^Parameter uncertainty contributes to the tardiness of starts of to-follow cases from their scheduled start times [1,2] because these algorithms rely on knowledge of the mean and variance of duration (e.g., in the log scale) of the procedure(s) for each case

Attribute	Explanation	Implications
Surgery is non-preemptive^a^	Once a procedure starts, it cannot generally be interrupted and restarted on another day	Add-on cases will often result in over-utilized hours because they will start after the scheduled list of cases in a suitable room (i.e., with appropriate nursing and anesthesia staffing)
There is variability in case duration due to process variation	Process variation occurs in surgical duration due to patient-related factors such as anatomical differences, the extent of disease, and technical surgical challenges [23]	Different patients having the same procedure will have different surgical durations
Many cases consist of a procedure or a combination of procedures that surgeons perform infrequently^b^	There are many different combinations of surgeons and procedures, and case duration varies among surgeons doing the same procedure(s)	Case duration prediction needs to be by surgeon and a combination of procedures
		There are many surgeon preference cards
		There is a lack of standardization of supplies
		There is a need for specialized nursing and anesthesia teams, constraining the ability to move cases
		Small sample sizes and resulting parameter uncertainty^c^ lead to wide prediction intervals for case durations

Identification of pediatric hospitals

Among the 718 non-federal facilities in the database, we identified the six pediatric hospitals in the state that reported to AHCA under their own facility number. Our screening criterion for identifying the pediatric hospitals was that at least 50% of the elective procedures were performed on patients <18 years of age. We manually verified that each facility identified was a pediatric hospital by inspecting their websites. We also searched the AHCA database for all facilities that included the string "child" or "pediatric" in their name and confirmed that we did not miss any pediatric hospitals. Pediatric hospitals that reported under an affiliated general hospital's facility number were not included because we could not determine reliably at which location surgery occurred in patients <18 years of age (e.g., Holtz Children's Hospital, reporting under the facility number of Jackson Memorial Hospital). Nicklaus Children's Hospital and the Nicklaus Children's Ambulatory Surgery Center, part of the Nicklaus Children's Health System, report their cases under two separate facility numbers for historical reasons; thus, we combined those two facilities as representing a pediatric hospital.

Identification of pediatric surgeons

The AHCA database includes the national provider identifier (NPI) of the performing provider for the primary procedure. We classified a provider as a pediatric surgeon if they performed surgery at a pediatric hospital or if >50% of their cases involved patients aged <18 years.

Identification of elective surgical cases

For ambulatory patients, an encounter was counted as involving elective surgery if it was performed on a regular workday, if there were non-zero intraoperative work relative value units (wRVUs) associated with any of the CPT codes performed during the encounter, and if there were non-zero American Society of Anesthesiologists base units associated with the principal CPT code (i.e., having the largest number of wRVUs).

For inpatients, an admission was counted as involving elective surgery if the date of the primary procedure associated with the admission was on the date of admission and was performed on a regular workday, if the ICD-10 code was for a procedure classified as major therapeutic or major diagnostic, if the admission was not listed as urgent or emergent, and if there were no emergency department charges for the admission.

Determination of surgeons' average daily caseload

For each of the six pediatric hospitals, we calculated the percentage of their lists with either one or two elective cases [16]. We also calculated the mean percentage among all facilities [24]. To account for the correlation of lists within facilities among surgeons, for each hospital, we calculated the proportion of the hospital’s lists with one or two cases as the number of lists containing one or two cases divided by the hospital’s total number of lists [16]. The Freeman-Tukey transformation was then applied to each hospital’s proportion [25]. The Student's t-distribution was used to calculate 99% CIs for the transformed values among hospitals [24]. Using the harmonic mean of the number of lists at each hospital (appropriate because we were computing the mean of ratios), we then applied back-transformation to determine the 99% CI of the mean percentage [25,26]. The Freeman-Tukey transformed percentages of lists with one or two cases were compared pairwise with 50% transformed based on the total sample size using Student's one-group two-sided t-test, thereby determining if most surgeons' lists (i.e., more than 50%) comprised only one or two cases.

Determination of surgeons’ average weekly caseload in 2018 and 2019

We identified each surgeon who operated at a pediatric hospital in 2019. Then, for 2018, we calculated each of these surgeons' average weekly caseload at that hospital during each four-week interval where they operated on at least one patient, as follows. First, for each surgeon-hospital combination, we determined the number of regular workdays in each of the 13 four-week intervals in 2018 during which the surgeon operated on at least one patient at that hospital. We then divided the total number of cases done in 2018 by the surgeon at the hospital by the total number of regular workdays (i.e., excluding the six yearly federal holidays observed statewide) during those four-week intervals; we multiplied this ratio by 5 to get the average weekly caseload [16]. If a surgeon was present in 2019 but not in 2018, they were assigned an average caseload of zero in 2018 (e.g., the surgeon joined the hospital in 2019). If the surgeon was present in 2018 but not in 2019, their caseload in 2019 was assigned a value of zero.

Calculation of growth at pediatric hospitals from 2018 to 2019

We analyzed the increase in elective cases done by each surgeon working at a pediatric hospital from 2018 to 2019, setting growth = 0 if there was a decline. Of the six pediatric hospitals, one hospital was excluded because it stopped doing surgery in July 2019, thereby preventing the assessment of growth. Overall growth at a hospital was calculated as the sum of the surgeons’ growth at that hospital. Surgeons were classified as low-caseload (average weekly caseload of ≤2 cases per week) or moderate-high caseload (>2 cases per week). The fractional contribution to growth among low-caseload surgeons was the sum of the growth among all surgeons in this group divided by the overall growth at the hospital.

Calculation of growth in wRVU at each pediatric hospital from 2018 to 2019

For the two surgeon groups (low and moderate-high caseload), we determined their contribution to growth in the number of wRVUs at the hospital, assigned only for ambulatory cases. For each case, we first sorted the listed procedures in descending order by wRVU. Total wRVUs were calculated as the sum of the highest wRVU (i.e., the principal procedure) and 50% of the sum of the next four procedures’ wRVU. Growth was then determined for wRVU as described below.

Statistical analysis of growth in caseload and growth in wRVUs

The statistical methods followed those described in our previous surgical growth study from Iowa [17].

Firstly, for each of the two surgeon groups at the five pediatric hospitals, we determined their contribution to growth in the surgical caseload at the hospital. Growth was measured for each surgeon as the maximum of zero and the change in their caseload from 2018 to 2019 at the hospital (i.e., if caseload decreased, growth was zero). Overall growth at a hospital was calculated as the sum of the surgeons’ growth at that hospital. The fractional contribution to growth among surgeons in each group was the sum of the growth among all surgeons in the group divided by the overall growth at the hospital. The fractional growths among the two groups summed up to 100% because the groups were non-overlapping, every surgeon was included in a group, and every hospital studied did outpatient surgery (i.e., there were no hospitals where every case's wRVU was zero).

Secondly, we calculated the mean and standard error of these five fractions for caseload growth and compared the mean with 50% using the two-sided one-group Student’s t-test and a confidence level of 99% (alpha = 0.01).

Finally, for the two surgeon groups (low and moderate-high caseload), we determined their contribution to growth in the number of wRVUs at the hospital, assigned only for ambulatory cases. For each case, we first sorted the listed procedures in descending order by wRVU. Total wRVUs were calculated as the sum of the highest wRVU (i.e., the principal procedure) and 50% of the sum of the case's next four procedures’ wRVUs, if listed. The fractional contribution to growth from the two surgeon groups was then determined as described for the fractional growth in caseload.

All calculations were performed with R Studio v1.2.1335 (RStudio, Boston, MA) under R version 4.0.3 (R Foundation for Statistical Computing, Vienna, Austria). We did not perform a power analysis because we included all pediatric hospitals in the state (i.e., our dataset was not a sample, but rather included the entire population).

## Results

Among the 72,293 elective cases at the six pediatric hospitals in Florida in 2018 and 2019, 71,340 were studied, following various exclusions (Table 2). Of these cases, 63,870 (88.2%) were for ambulatory surgery and 8,423 (11.8%) for inpatient surgery (Table 2). There were 27,557 lists of surgeon, hospital, and date and 19,232 lists of surgeon, hospital, and week (Table 2). There were 817 surgeons who operated at the pediatric hospitals.

**Table 2:** Cases analyzed at pediatric hospitals in Florida among all elective cases ^a^The number of combinations of surgeons performing at least one elective case at a hospital on a specific date. For example, a surgeon doing elective cases at a specific hospital on 30 regular workdays would contribute 30 lists to the total. The number of cases performed on each date does not contribute to the number of lists ^b^The number of combinations of surgeons performing elective surgery at a hospital during a specific week. For example, a surgeon doing elective cases at a specific hospital on any regular workday for 15 weeks would contribute 15 lists to the total. For example, if a surgeon operated on Monday and Wednesday during a week, that would count as one list NPI: national provider identifier

Elective case description	Ambulatory	Inpatient	Total
Total cases	63,870	8,423	72,293
Weekend cases, excluded	-689	-19	-708
Holiday cases, excluded	-33	-1	-34
Regular workday cases, missing or invalid NPI	-205	-6	-211
Elective cases analyzed (n = 71,340)	62,943	8,397	71,340
Percentage of elective cases analyzed	88.2%	11.8%	100%
Number of lists of surgeon, hospital, and date^a^			27,557
Number of lists of surgeon, hospital, and week^b^			19,232

Frequency of surgeon lists having one or two elective cases at the six pediatric hospitals

**Figure 1:**
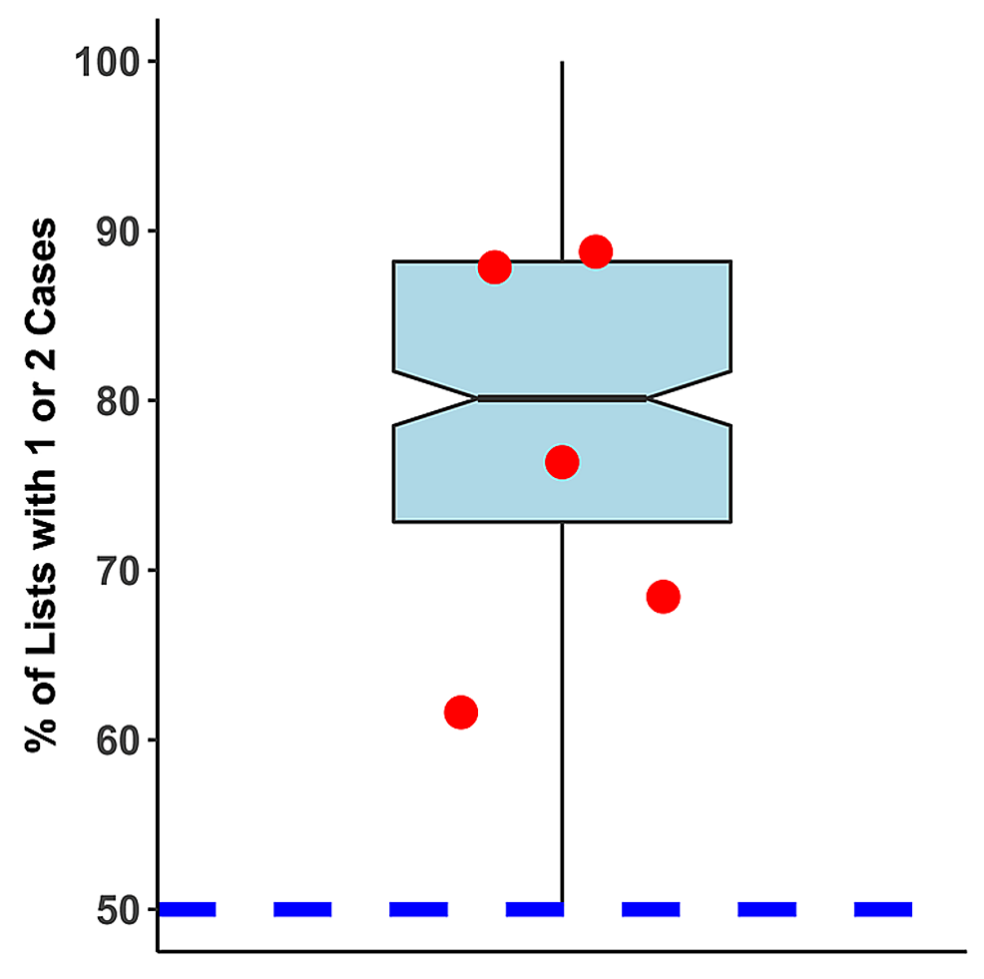
Percentage of lists (i.e., combinations of surgeon, hospital, and date) comprising one or two cases The box plot represents the distribution of such lists among the 455 non-pediatric hospitals in the state, whereas the red circles represent the five studied pediatric hospitals. The box edges represent the first and third quartile and the whiskers 1.5x the interquartile range. The middle line in the box is the median, and the notches the standard error of the median. The blue dotted line represents 50% of lists containing one or two cases

Averaging among the six pediatric hospitals, the non-holiday, weekday lists of elective cases of most surgeons at each facility had just one or two cases (68.1%, Table 3). All six hospitals had fractions of cases with just one or two cases >50% (p = 0.016 compared to 50%). Also averaging among these hospitals, many lists comprised a single case (47.6%, Table 3). Overall, the percentage of lists with one or two elective cases at the pediatric hospitals was similar to that found at non-pediatric hospitals in the state (Figure 1).

When surgeons' lists of cases were calculated by week rather than by day (i.e., simulating a theoretical consolidation of surgeons operating on multiple days each week to a single day), most lists would still comprise one or two cases (mean: 61.7%, Table 3). All six hospitals, when calculated by week, had fractions of cases with just one or two cases >50% (p = 0.016 compared to 50%).

Pooling among all six hospitals, the percentages of daily lists (n = 27,557) with one or two cases was 66.9% (99% CI: 66.2% to 67.6%) and with only one case was 47.9% (99% CI: 47.1% to 48.7%). Similarly, the percentages of weekly lists (n = 19,232) with one or two cases were 65.1% (99% CI: 64.8% to 65.5%) and with only one case was 43.9% (99% CI: 43.6% to 44.2%).

**Table 3:** Frequency, statewide, of surgeons' lists containing just one or two cases among the six pediatric hospitals in Florida (mean, 99% confidence interval) ^a^The total number of the daily lists was 27,557, and the harmonic mean was 2,428.2 ^b^The total number of the weekly lists was 19232, and the harmonic mean was 1,480.8 ^c ^Using the binomial test and comparing to 50%, all six hospital's fractions of lists with one or two cases were >0.5, one-sided p = 0.016. The one-sided p-value is justified because we expected that the value should be >0.5 from the previous study and we were testing to confirm ^d^Using the two-sided one-group Student's t-test, comparing to 50%, p = 0.012 ^e^Using the two-sided one-group Student's t-test, comparing to 50%, p = 0.049

Criteria of list	By workday^a^	By week^b^
Average hospital's percentage of surgeon-day combinations with one or two cases	68.1% (49.1% to 84.3%)^c,d^	61.7% (43.45 to 47.4%)^c,e^
Average hospital's percentage of surgeon-day combinations with one case	47.6% (35.9% to 59.4%)	35.6% (17.5% to 56.2%)
Surgeon, hospital, and day combinations with one or two cases, pooled among all hospitals	66.9% (66.2% to 67.6%)	65.1% (64.8% to 65.5%)
Surgeon, hospital, and day combinations with one case, pooled among all hospitals	47.9% (47.1% to 48.7%)	43.9% (43.6% to 44.2%)

Frequency of lists performed by pediatric surgeons containing one or two cases at non-pediatric hospitals

Pooling among the 411 surgeons working at non-pediatric hospitals whose pediatric cases (i.e., age <18 years) represented >75% of their total caseload, 54.7% (99% CI: 53.8% to 55.5%, p<0.0001 vs. 50%) of their daily lists (n = 24,579) comprised one or two cases, and 35.8% had one case. Among surgeons working at non-pediatric hospitals whose pediatric caseload was >50% of their total caseload, 58.3% (99% CI: 57.3% to 58.7%, p<0.0001 vs. 50%) of their lists comprised one or two cases and 39.0% of their lists had one case. These values were less than the corresponding values for the surgeons working at pediatric hospitals: 66.9% and 47.9% for one or two cases, or one case, respectively.

Surgical growth at pediatric hospitals

Growth in surgical caseload at the pediatric hospitals between 2018 and 2019 was mostly attributable to surgeons who in 2018 averaged ≤2.0 cases per week (n = 5 hospitals, 76.3%% ± 5.4%, p = 0.0085 compared to 50%, Table 4). Similarly, growth in wRVUs was also mostly attributable to these low-caseload surgeons (73.8% ± 5.4%, p = 0.017 compared to 50%, Table 4).

**Table 4:** Surgical growth between 2018 and 2019 among surgeons working at the five studied pediatric hospitals in Florida ^a^Two-sided one-group Student's t-test comparing the contribution of growth in 2019 to 50% ^b^The sum of the contribution to growth in caseload among the five studied hospitals for surgeons performing ≤2.0 cases per week and those performing >2.0 cases per week = 100%. This was a consequence of every studied hospital having growth in caseload ^c^The sum of the contribution to growth in wRVU among the 202 studied hospitals for surgeons performing ≤2.0 cases per week and those performing >2.0 cases per week = 100%. This was a consequence of every studied hospital having growth in wRVU SE: standard error; wRVU: intraoperative work relative value units

Growth parameter	Surgeons' weekly caseload in 2018	Contribution to growth in 2019 (mean ± SE)	P-value compared to 50%^a^
Caseload^b^	≤2.0	76.3% ± 5.4%	0.0085
	>2.0	23.7% ± 5.4%	0.0085
wRVU^c^	≤2.0	73.8% ± 6.1%	0.017
	>2.0	26.2% ± 6.1%	0.017

## Discussion

Most pediatric surgeons' lists of cases in Florida comprised only one or two cases, and most of the surgical growth at pediatric hospitals accrued from the combined workload of these low-caseload surgeons. These results are consistent with our previous studies of surgeon lists and surgical growth at the predominantly adult hospitals in the state [16,17].

The total number of pediatric hospitals in the state (n = 5) was too small to validly compare the quantitative values obtained from the pediatric surgeons to non-pediatric surgeons (i.e., the CIs are too large); thus, we cannot comment if there are true differences between adult and pediatric hospitals. However, the fractions of lists with only one or two cases are similar (Figure 1). Nonetheless, for both types of hospitals, the message is clear: hospitals need to ensure that low-caseload surgeons have access to the OR schedule if they want to facilitate growth in surgical caseload. This conclusion holds despite inherent differences in surgical procedures performed in children vs. adults and their much lesser degree of comorbidities, overall.

Because very few low-caseload surgeons, by definition, would be eligible for block time on an individual basis [14,22], hospital administrators need to ensure that sufficient allocated time (i.e., time staffed by nursing and anesthesia) is provided so that these surgeons can perform their cases [21]. A consequence of not having block time is that low-caseload surgeons will often be performing their elective cases in the afternoon [27,28]; thus, one afternoon, weekly or biweekly, likely would need to be set aside [22], depending on how long the surgeons are willing to wait once a decision to operate has been made. Finally, administrators need to ensure that if a low-caseload surgeon schedules cases into the allocated time of another service (i.e., the service projected to have the most unused time), the time is released sufficiently early to allow for the management of the logistics of case scheduling on relatively short notice (e.g., office schedule changes, securing insurance authorization, coordination with patients) [21,29].

Our study provides additional support as the quantitative OR management methods developed primarily using data from adult hospitals apply equally to pediatric hospitals (Table 1). The fundamental attributes of adult or pediatric surgery are not different. If anything, there is more uncertainty in the accuracy of case duration prediction for pediatric surgery [9].

Limitations

Our study had too few pediatric hospitals (i.e., insufficient statistical power) to compare growth at the pediatric hospitals to that at the adult hospitals. Nonetheless, the same general finding was found: growth in caseload was mostly due to surgeons who operated on only one or two cases per week. There are several pediatric hospitals in the state that we could not include because they report their cases to AHCA under the facility number of the affiliated adult hospital system. Although patient age was included in the database, we had no way of knowing accurately if a patient aged <18 years was operated on at the pediatric or the adult hospital. As a sensitivity analysis, we looked at surgeons who operated at non-pediatric hospitals in the state whose practices were predominantly among patients <18 years of age and found similar results for short surgeon lists. However, we could only measure growth in pediatric hospitals as those facilities are homogeneous regarding the presence of pediatric patients.

## Conclusions

As is applicable to adult hospitals, most pediatric surgeons’ lists of cases comprise only one or two cases per day, with many lists having a single case. Growth at pediatric hospitals accrued from low-caseload surgeons performing one or two cases per week in the preceding year. These findings imply that hospitals desiring to increase their surgical caseload should ensure that low-caseload surgeons have access to the OR schedule. Furthermore, because neither raw nor adjusted percent utilization can be measured accurately for low-caseload surgeons, these metrics should not be used to allocate OR time to individual surgeons. Because most adult and pediatric surgeons have low caseloads, this is a fundamental attribute of surgery.

## References

[1] Dexter F, Dexter EU, Ledolter J (2010). Influence of procedure classification on process variability and parameter uncertainty of surgical case durations. Anesth Analg.

[2] Dexter F, Bayman EO, Pattillo JCS, Schwenk ES, Epstein RH (2018). Influence of parameter uncertainty on the tardiness of the start of a surgical case following a preceding surgical case performed by a different surgeon. Perioper Care Oper Room Manag.

[3] Dexter F, Macario A (2000). What is the relative frequency of uncommon ambulatory surgery procedures performed in the United States with an anesthesia provider?. Anesth Analg.

[4] Dexter F, Traub RD, Fleisher LA, Rock P (2002). What sample sizes are required for pooling surgical case durations among facilities to decrease the incidence of procedures with little historical data?. Anesthesiology.

[5] O'Neill L, Dexter F, Park SH, Epstein RH (2017). Uncommon combinations of ICD10-PCS or ICD-9-CM operative procedure codes account for most inpatient surgery at half of Texas hospitals. J Clin Anesth.

[6] Zhou J, Dexter F, Macario A, Lubarsky DA (1999). Relying solely on historical surgical times to estimate accurately future surgical times is unlikely to reduce the average length of time cases finish late. J Clin Anesth.

[7] Dexter F, Traub RD (2000). Sequencing cases in the operating room: predicting whether one surgical case will last longer than another. Anesth Analg.

[8] Dexter F, Epstein RH, Penning DH (2020). Late first-case of the day starts do not cause greater minutes of over-utilized time at an endoscopy suite with 8-hour workdays and late running rooms. A historical cohort study. J Clin Anesth.

[9] Bravo F, Levi R, Ferrari LR, McManus ML (2015). The nature and sources of variability in pediatric surgical case duration. Paediatr Anaesth.

[10] Jiao Y, Sharma A, Ben Abdallah A, Maddox TM, Kannampallil T (2020). Probabilistic forecasting of surgical case duration using machine learning: model development and validation. J Am Med Inform Assoc.

[11] Dexter F, Ledolter J (2005). Bayesian prediction bounds and comparisons of operating room times even for procedures with few or no historic data. Anesthesiology.

[12] Dexter F, Epstein RH, Lee JD, Ledolter J (2009). Automatic updating of times remaining in surgical cases using Bayesian analysis of historical case duration data and "instant messaging" updates from anesthesia providers. Anesth Analg.

[13] Dexter F, Macario A, Traub RD, Hopwood M, Lubarsky DA (1999). An operating room scheduling strategy to maximize the use of operating room block time: computer simulation of patient scheduling and survey of patients' preferences for surgical waiting time. Anesth Analg.

[14] Dexter F, Macario A, Traub RD, Lubarsky DA (2003). Operating room utilization alone is not an accurate metric for the allocation of operating room block time to individual surgeons with low caseloads. Anesthesiology.

[15] Dexter F, Jarvie C, Epstein RH (2017). At most hospitals in the state of Iowa, most surgeons' daily lists of elective cases include only 1 or 2 cases: individual surgeons' percentage operating room utilization is a consistently unreliable metric. J Clin Anesth.

[16] Epstein RH, Dexter F, Fahy BG, Diez C (2021). Most surgeons' daily elective lists in Florida comprise only 1 or 2 elective cases, making percent utilization unreliable for planning individual surgeons' block time. J Clin Anesth.

[17] Dexter F, Jarvie C, Epstein RH (2018). Lack of generalizability of observational studies' findings for turnover time reduction and growth in surgery based on the State of Iowa, where from one year to the next, most growth was attributable to surgeons performing only a few cases per week. J Clin Anesth.

[18] Epstein RH, Dexter F, Diez C, Fahy BG (2021). Elective surgery growth at Florida hospitals accrues mostly from surgeons averaging 2 or fewer cases per week: a retrospective cohort study [PREPRINT]. J Clin Anesth.

[19] Boggs SD, Tsai MH, Urman RD (2018). The Association of Anesthesia Clinical Directors (AACD) glossary of times used for scheduling and monitoring of diagnostic and therapeutic procedures. J Med Syst.

[20] Strum DP, Vargas LG, May JH, Bashein G (1997). Surgical suite utilization and capacity planning: a minimal cost analysis model. J Med Syst.

[21] McIntosh C, Dexter F, Epstein RH (2006). The impact of service-specific staffing, case scheduling, turnovers, and first-case starts on anesthesia group and operating room productivity: a tutorial using data from an Australian hospital. Anesth Analg.

[22] Dexter F, Epstein RH, Podgorski EM 3rd, Pearson AC (2020). Appropriate operating room time allocations and half-day block time for low caseload proceduralists, including anesthesiologist pain medicine physicians in the State of Florida. J Clin Anesth.

[23] Dexter F, Dexter EU, Masursky D, Nussmeier NA (2008). Systematic review of general thoracic surgery articles to identify predictors of operating room case durations. Anesth Analg.

[24] Dexter F, Marcon E, Epstein RH, Ledolter J (2005). Validation of statistical methods to compare cancellation rates on the day of surgery. Anesth Analg.

[25] Miller JJ (1978). The inverse of the Freeman-Tukey double arcsine transformation. Am Stat.

[26] Dexter F, Abouleish A, Marian AA, Epstein RH (2021). The anesthetizing sites supervised to anesthesiologist ratio is an invalid surrogate for group productivity in academic anesthesia departments when used without consideration of the corresponding managerial decisions. J Clin Anesth.

[27] Sulecki L, Dexter F, Zura A, Saager L, Epstein RH (2012). Lack of value of scheduling processes to move cases from a heavily used main campus to other facilities within a health care system. Anesth Analg.

[28] Wachtel RE, Dexter F (2009). Influence of the operating room schedule on tardiness from scheduled start times. Anesth Analg.

[29] Dexter F, Traub RD, Macario A (2003). How to release allocated operating room time to increase efficiency: predicting which surgical service will have the most underutilized operating room time. Anesth Analg.

